# Supplementary material for: CellCycleTRACER accounts for cell cycle and volume in mass cytometry data
Source: Nat Commun. 2018 Feb 12;9:632. doi: 10.1038/s41467-018-03005-5 (PMC5809393; doi:10.1038/s41467-018-03005-5)
Supplement: Supplementary file 1 — Supplementary Information [file 41467_2018_3005_MOESM1_ESM.pdf]

Supplementary Information

Rapsomaniki et al., CellCycleTRACER Accounts for Cell Cycle and Volume in Mass Cytometry Data

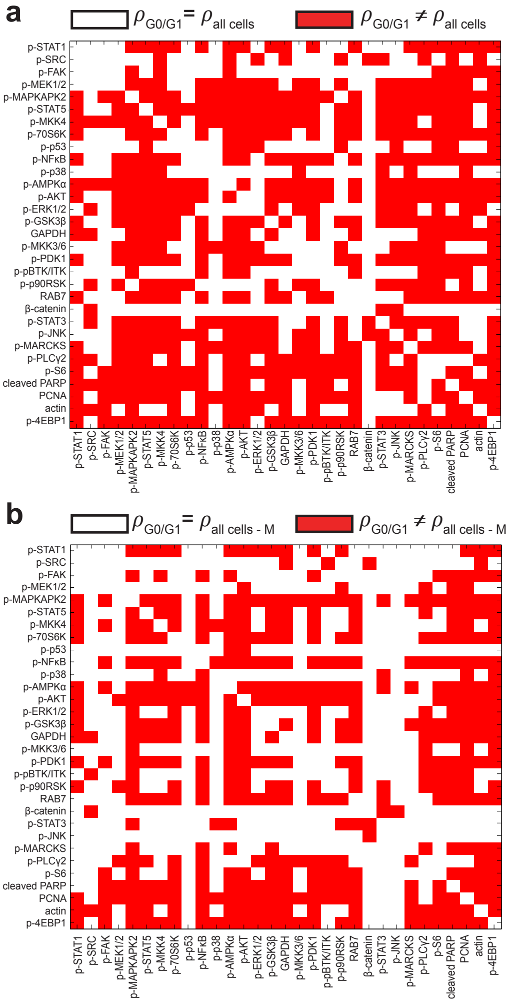

Supplementary Figure 1: **Pairwise correlation coefficients as computed from a mixed population of THP-1 cells are significantly influenced by the cell cycle state.** **(a)** Comparison of Spearman correlation coefficients computed using only G0/G1 cells, $\rho_{\mathrm{G0/G1}}$, and using all cells, $\rho_{\text{all cells}}$ (details in Supplementary Note 1) indicates that the majority of protein pairs (292 out of total 465) exhibit statistically significant changes in correlation across the cell cycle (p-value $\leq$ 0.05). **(b)** Excluding M phase cells from the data and repeating the analysis reveals a minor improvement in the introduced bias; however significant changes in the correlation coefficients are still observed in almost half of the protein pairs (222 out of 465).

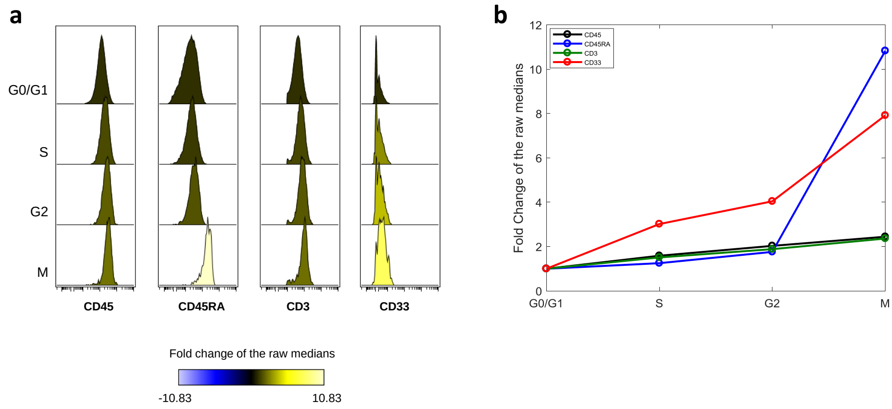

Supplementary Figure 2: **Cell cycle effects in cell surface markers.** To assess the effect of the cell cycle in a dataset that includes cell membrane markers, we analyzed the data from [1], where the authors used mass cytometry to analyze a population of human T cells and included in their panel cell surface markers (e.g., CD3, CD4, CD45) as well as cell cycle markers (e.g., p-HH3, p-RB, IdU, cyclin B1). When examining the distribution of the cell surface markers across the cell cycle phases, as identified by the authors via manual gating, we observed fluctuations in levels across the cell cycle for some of the measured markers **(a)**. Specifically, CD45, CD45RA, CD3, and CD33 progressively increase during the entire cell cycle, peaking at the M phase (2.5- to 11-fold increase with respect to G0/G1 phase **(b)**). Overall, this finding indicates that, even in well-studied systems where the cell cycle is not expected to be a prominent confounder, cell-cycle signatures can have a non-negligible imprint on the measured protein abundance.

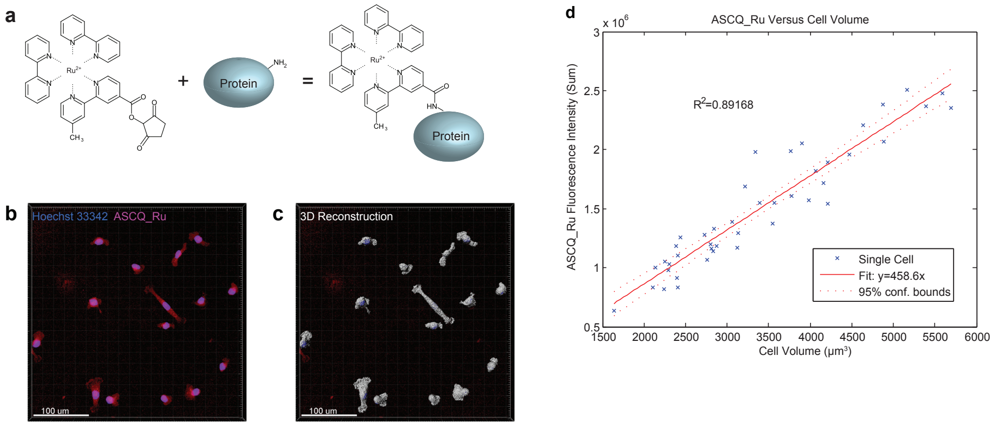

Supplementary Figure 3: **Validation of ASCQ_Ru as an indicator of cell volume.** **(a)** Structure of ASCQ_Ru and biochemical mechanism of ASCQ_Ru staining: ASCQ_Ru covalently binds to the amines on unspecific proteins. **(b)** Confocal images of MDA-MB-231 cells stained with ASCQ_Ru and Hoechst 33342 (DNA staining). **(c)** 3D reconstruction of imaged cells using ASCQ_Ru to determine cell volume. **(d)** Linear regression on summed ASCQ_Ru fluorescence intensity in single cells versus computed cell volume. Scale bar, 100μm.

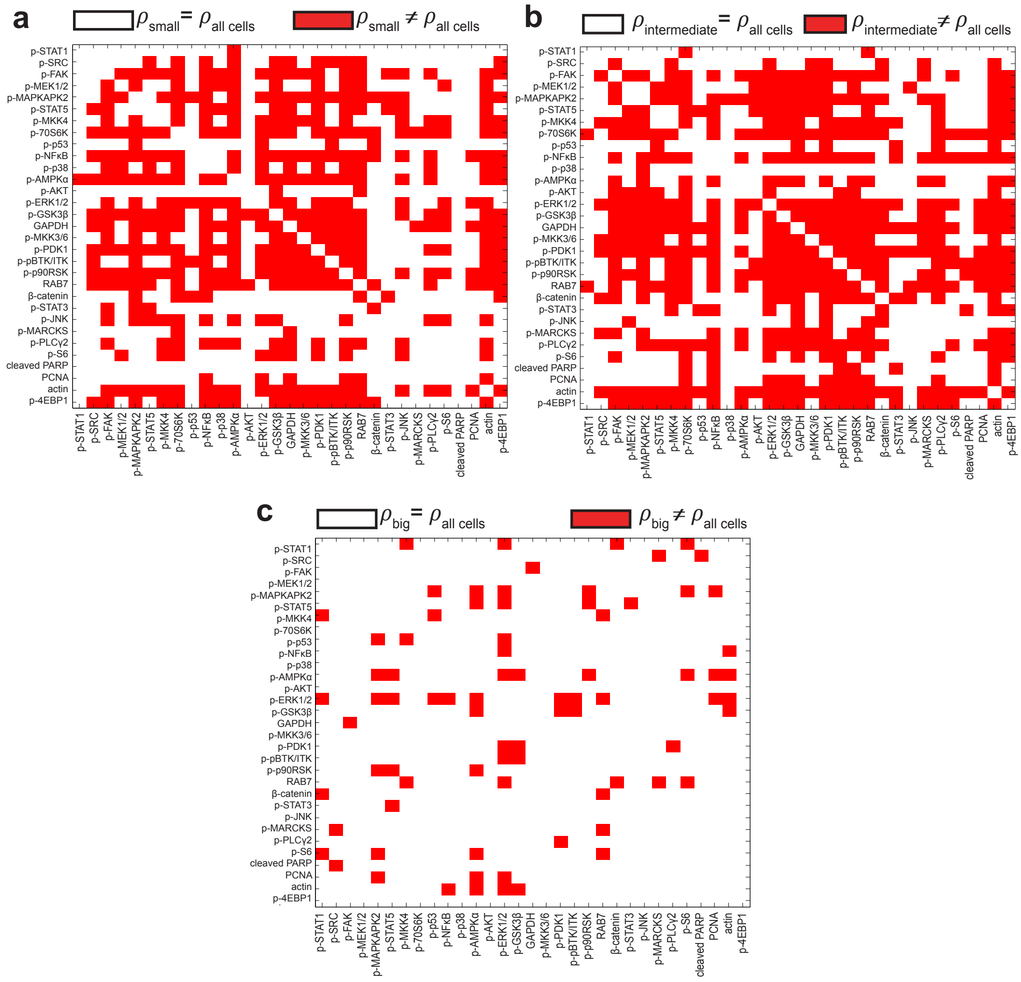

Supplementary Figure 4: **Pairwise correlation coefficients are significantly influenced by cell volume**. Pairwise correlations computed using the whole cell population are significantly different (p-value $\leq$ 0.05) than pairwise correlations computed using only **(a)** small cells (214 out of 465 pairs show statistically significant differences), **(b)** intermediate (247 out of 465 pairs) and big **(c)** (40 out of 465 pairs). The pairwise correlations are thus driven by large cells, and the cell volume can act as a confounding factor.

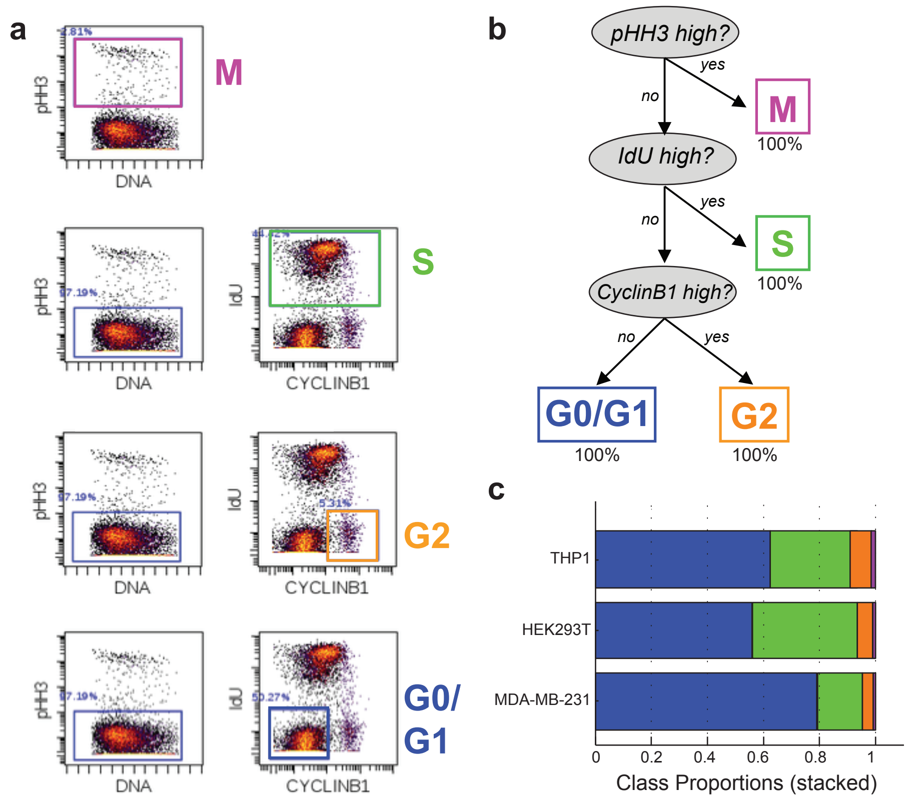

Supplementary Figure 5: **Cell cycle classification using decision trees.** **(a)** Measurements of the four cell cycle markers IdU, cyclin B1, p-HH3 and p-RB can be used for manual cell cycle phase assignment as described in [1]. Shown here are the results of this gating process for a THP-1 cell line, as performed in Cytobank. **(b)** Resulting structure of the decision tree after the training phase, with class labels and percentages indicated at the terminal nodes. The decision tree faithfully recapitulates the class assignment performed by manually gating, both in terms of the markers selected per class and the order of selection. **(c)** Cell cycle phase ratios in the three studied cell lines, as predicted by a decision tree trained on measurements of IdU, cyclin B1, p-HH3 and p-RB.

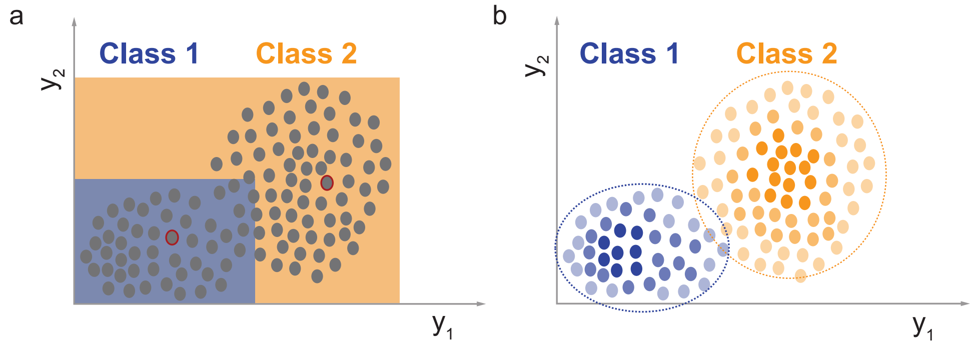

Supplementary Figure 6: **Decision trees and Gaussian mixture models enable automatic class assignment.** **(a)** Results of supervised learning with decision trees, in the space of hypothetical markers y_1, y_2 for two hypothetical classes 1 and 2. The decision boundaries are shown as areas with different colors. Class means (red circles), data covariance matrices and class proportions are used as initial parameters in a Gaussian mixture model with two components. **(b)** After fitting using the Expectation-Maximization (EM) [7] algorithm, the posterior probability that a single cell belongs to each class is returned and the class with the higher posterior is selected. Here, color indicates class assignment and opacity indicates posterior probability value. Refined class means are indicated as red circles.

a Classification Performance of Decision Trees on HEK293T data

True Class \ Predicted Class	G0/G1	S	G2	M	
G0/G1	8240	0	0	12	99.8%
S	96	7662	49	0	98.1%
G2	82	0	618	0	88.3%
M	0	8	0	308	97.5%

b Results after Prediction Refinement with GMMs

True Class \ Predicted Class	G0/G1	S	G2	M	
G0/G1	8240	0	0	12	99.8%
S	24	7702	81	0	98.7%
G2	14	0	686	0	98.3%
M	0	4	0	312	98.7%

Supplementary Figure 7: **The Gaussian Mixture Model improves cell cycle classification results of decision trees.** **(a)** Confusion matrix of the classification performance on an independent test set from HEK293T data after prediction using decision trees. **(b)** Final confusion matrix after classification refinement by fitting a Gaussian mixture model of four components to the same HEK293T data. The results indicate an overall improvement of the prediction across all classes when decision trees and GMMs are combined.

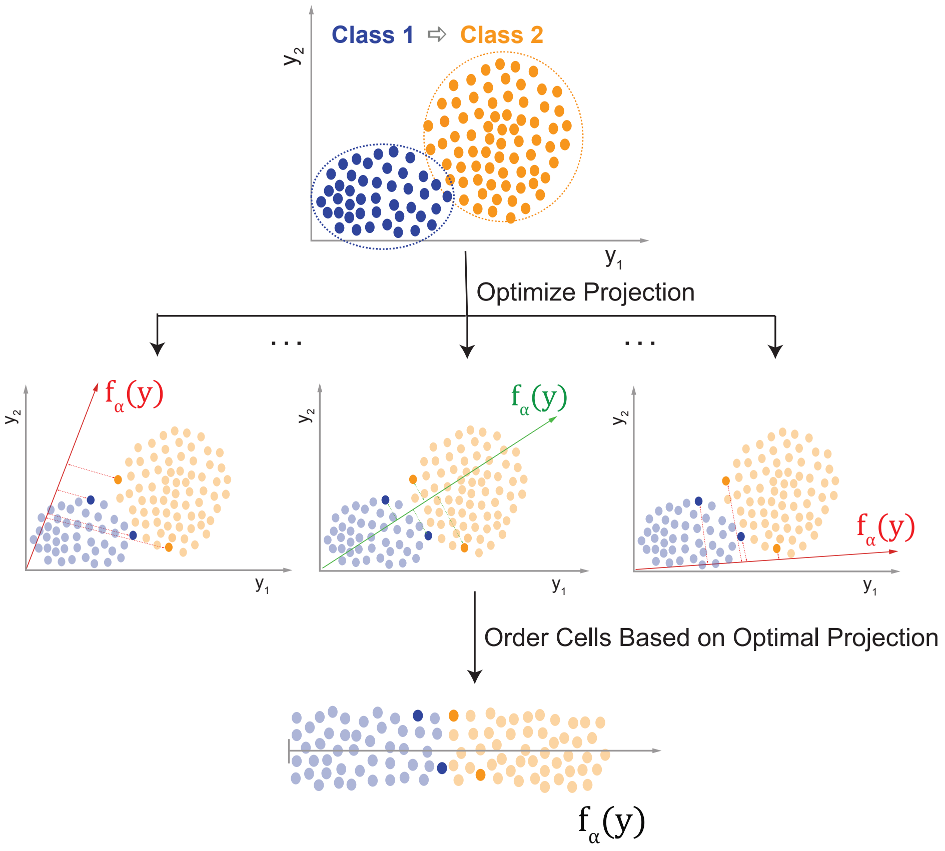

Supplementary Figure 8: **Outline of the trajectory reconstruction method.** Toy example of the trajectory reconstruction method for two classes in a two-dimensional space of hypothetical markers y_1, y_2. Given prior information about the single cell class labels and the class ordering, the best embedding $f_\alpha(y)$ is computed by selecting the function that optimally preserves the class ordering in the new subspace spanned by $f_\alpha(y)$. As class ordering violations are expected in the embedding due to noise or measurement outliers, we introduce slack variables, i.e. positive variables that penalize each constraint violation, and we minimize over the sum of all slack variables.

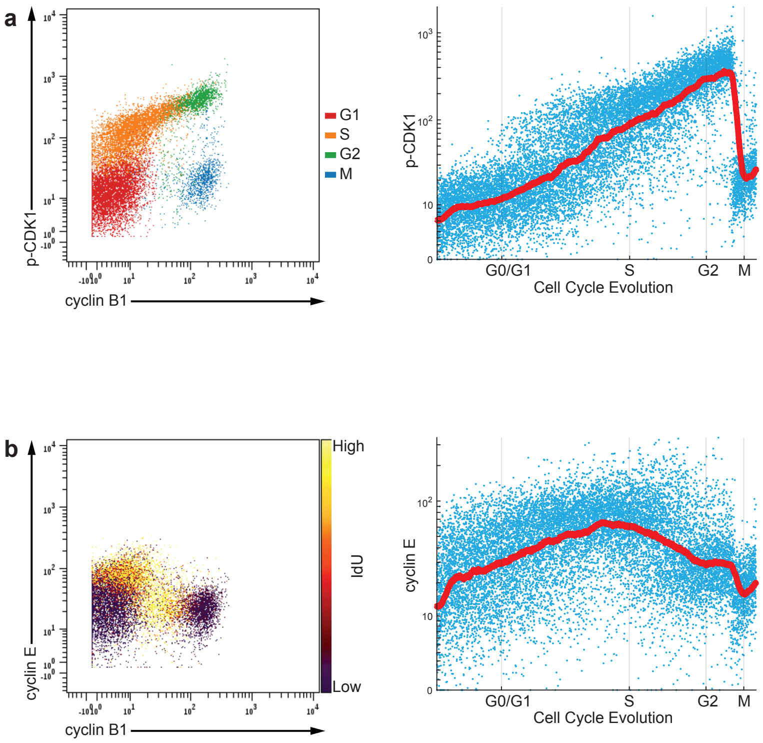

Supplementary Figure 9: **Cell cycle trajectories of p-CDK1 and cyclin E as independent validation.** **(a)** Phosphorylation of CDK1 on Tyr15 progressively increases during S and G2, peaks at the G2-M transition, and CDK1 is then dephosphorylated once cells enter M phase. Internal progressions of S phase and G2 phase are captured, and the G2-M transition is sharp. **(b)** At the same time, cyclin E progressively increases during G1, peaks at G1-S transition and degrades during S phase. In conclusion, the inferred cell cycle trajectories validate that our approach accurately captures S and G2 internal progression and the G2-M transition (p-CDK1), as well as the G1 phase progression and G1-S transition (cyclin E).

a

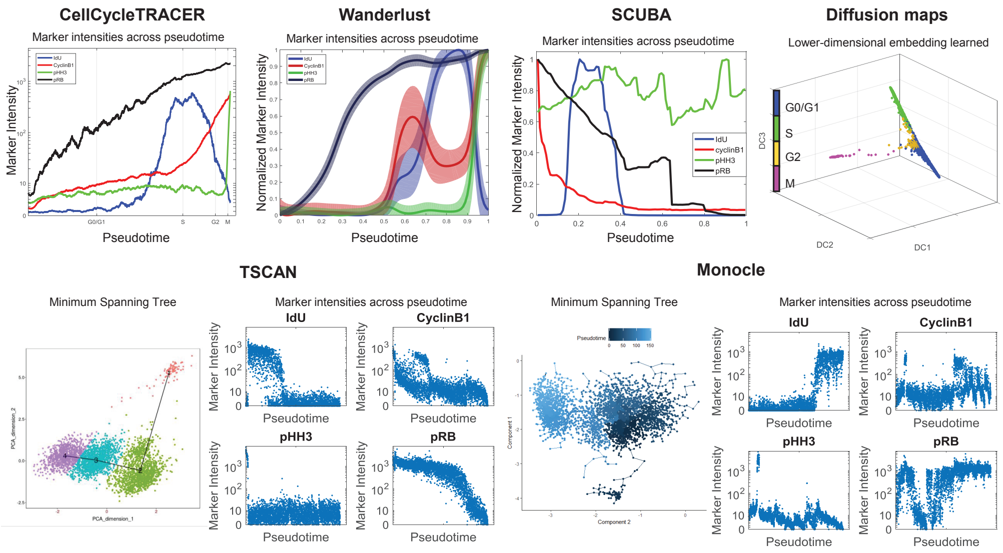

b

	Implementations	Runtime (in seconds)
CellCycleTRACER	MATLAB	9
Wanderlust	MATLAB (CYT implementation)	7
SCUBA	MATLAB (with drtoolbox and princurve packages)	627
Diffusion Maps	MATLAB (estimated σ = 0.16)	101
TSCAN	Bioconductor package	120
Monocle	Bioconductor package	1344

Supplementary Figure 10: **(a) Comparison of CellCycleTRACER with widely used trajectory reconstruction and embedding methods, as inferred from our four cell cycle marker measurements on a population of n =3753 THP-1 cells.** The cell cycle fluctuations of the markers do not represent the underlying biology. Wanderlust [2] ordered the cells in the wrong order (G1→G2→S→M). SCUBA [6] reconstructed a G2→S→G0/G1 trajectory and incorporated M phase cells in the other clusters. TSCAN [4] reconstructed a M→S/G2→G0/G1 trajectory by mixing together G2 and S cells, and Monocle [8] ordered the data as G0/G1→M→G0/G1→G2→S, by ordering M phase cells in the middle of the G0/G1 cluster. Last, diffusion maps [3] constructed a non-linear, low-dimensional embedding of the data, which did not capture the known ordering. **(b)** Implementation details and runtimes of the above-mentioned methods.

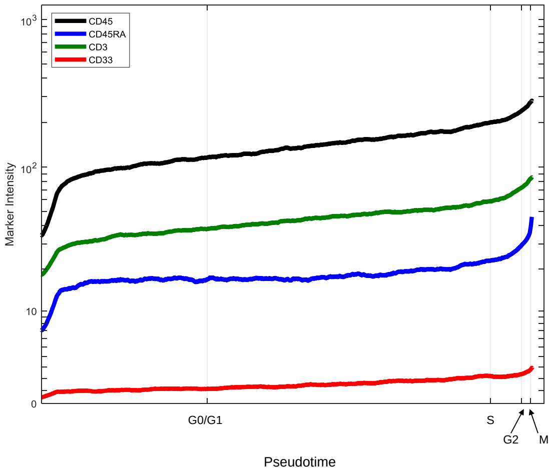

Supplementary Figure 11: **Cell cycle trajectories of cell surface markers.** Analysis of the published human T cell data [1] with CellCycleTRACER allowed the reconstruction of cell cycle trajectories of the cell surface markers. In agreement with the marker distributions across the cell cycle, shown in Supplementary Fig.2, the resulting trajectories for some of the surface proteins exhibited an increasing trend across the cell cycle, peaking as the cells enter M phase.

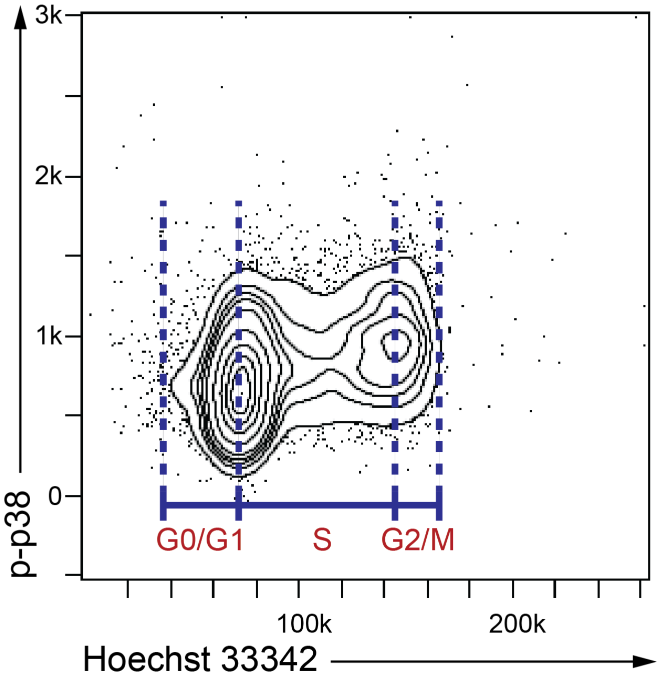

Supplementary Figure 12: **Cell-cycle-dependency of p38 phosphorylation in response to TNFα stimulation confirmed by flow cytometry.** THP-1 cells treated with TNFα for 15 min were measured by flow cytometry. A 1.5 fold increase of p-p38 level from the G0/G1 phase to G2/M phase can be observed, validating that the phosphorylation of p38 (Thr180/Tyr182) in response to TNFα stimulation is cell-cycle-dependent.

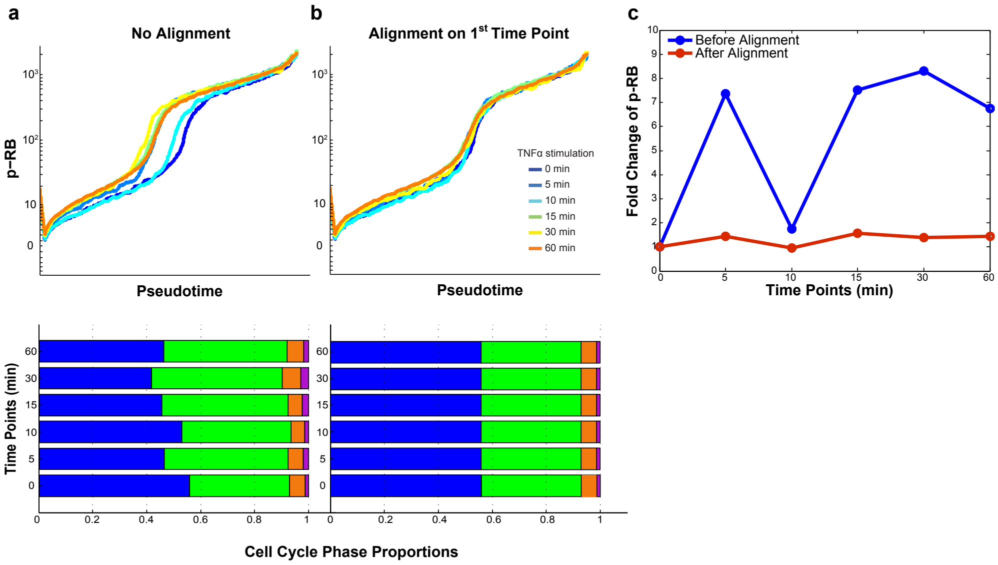

Supplementary Figure 13: **Trajectory alignment through cell cycle phase equalization removes duration-specific variability.** Trajectories of p-RB during TNFα stimulation in a population of HEK-293T cells, before and after cell cycle alignment. A larger population of S phase cells in time points 2, 4, 5, and 6 (**a**) results in relatively elevated levels of p-RB. When the cell cycle phases are aligned (**b**) no duration-specific variation is observed. Subsequently, estimating the fold change of the abundance of p-RB with respect to the control time point (**c**) results in large fold change values of time points 2, 4, 5, and 6; however, after equalizing the duration of the phases, p-RB levels are comparable across time points and the fold change is negligible.

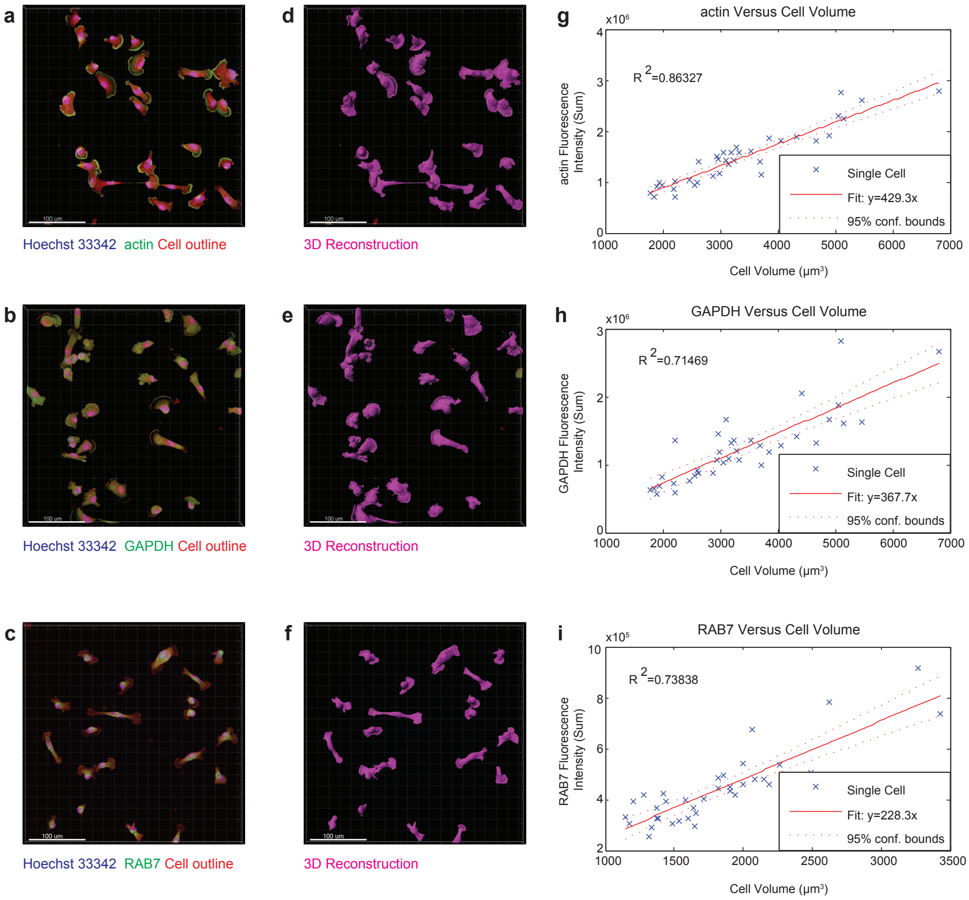

Supplementary Figure 14: **Validation of housekeeping proteins as a cell volume indicator.** **(a-c)** MDA-MB-231 cells were stained with Hoechst 33343 for the nucleus and Alexa Fluor 647 carboxylic acid succinimidyl ester for the cell outline. Housekeeping proteins actin, GAPDH and RAB7 were stained in (a), (b) and (c), respectively. **(d-f)** 3D reconstruction of corresponding images (a-c), using cell outline determined by Alexa Fluor 647 carboxylic acid succinimidyl ester. **(g-i)** Linear regression on the computed cell volume versus summed actin, GAPDH and RAB7 fluorescence intensity, respectively. Scale bar, 100μm.

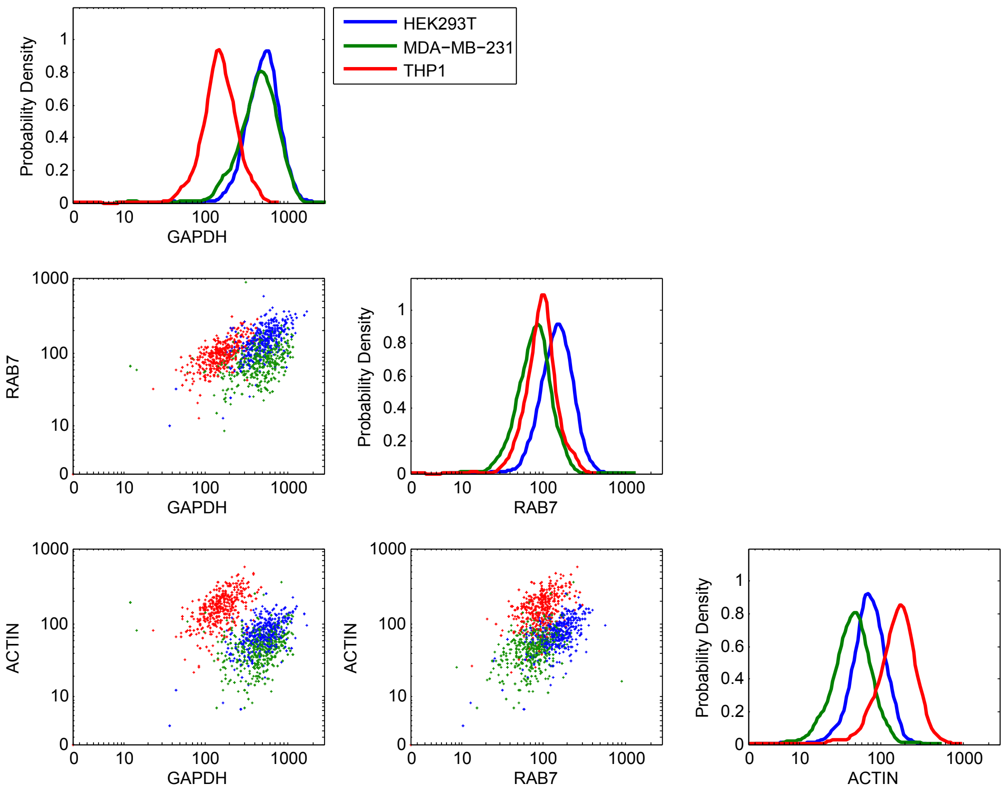

Supplementary Figure 15: **Housekeeping protein measurements across different cell lines reveal significant differences in protein abundances.** Probability density plots and pairwise scatter plots of the single cell measurements of housekeeping proteins GAPDH, RAB7 and actin in HEK293T, MDA-MB-231 and THP-1 cells show cell-line-specific variations in amounts of these proteins. For example, THP-1 cells have the lowest GAPDH levels but the highest actin levels. Thus, contrary to our expectations, housekeeping protein levels differ across cell types and are not optimal markers for cell volume correction.

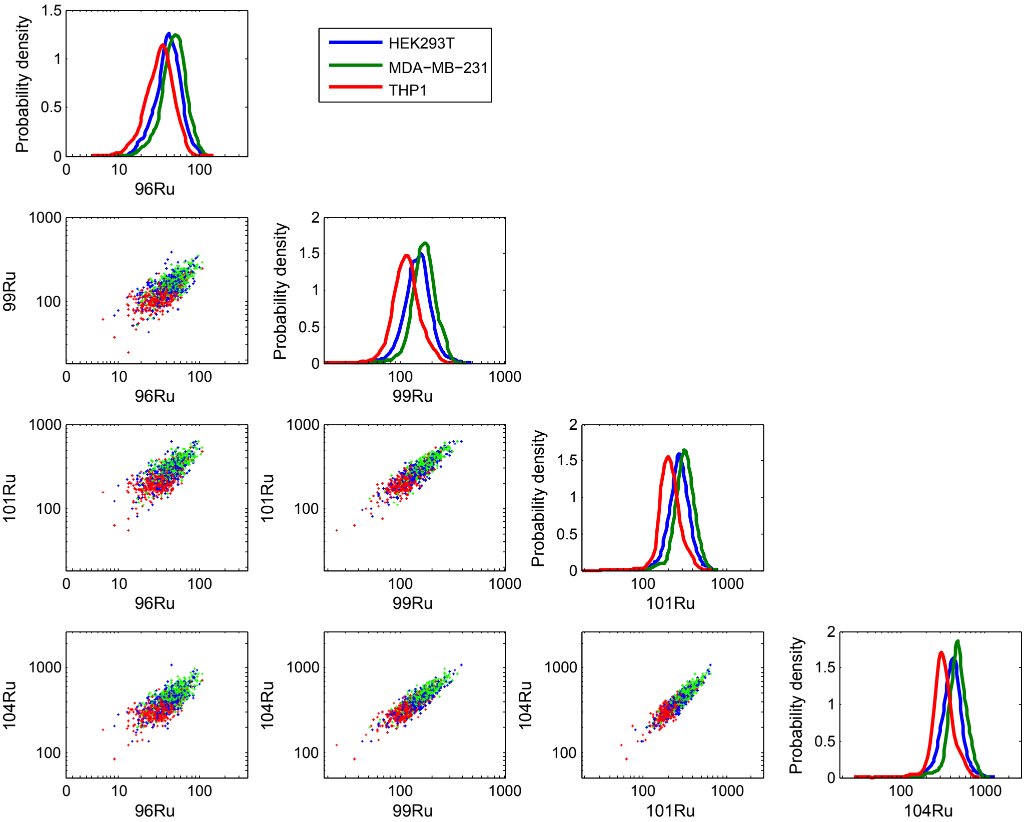

Supplementary Figure 16: **ASCQ Ru measurements are not affected by cell line variability.** Probability densities and pairwise scatter plots of the single cell measurements for four ruthenium stable isotopes, spanning all atomic masses, in HEK293T, MDA-MB-231 and THP-1 cells. In contrast to the housekeeping protein measurements, ASCQ_Ru is significantly more robust against cell-line-specific variations, allowing thus a more accurate quantification of cell volume.

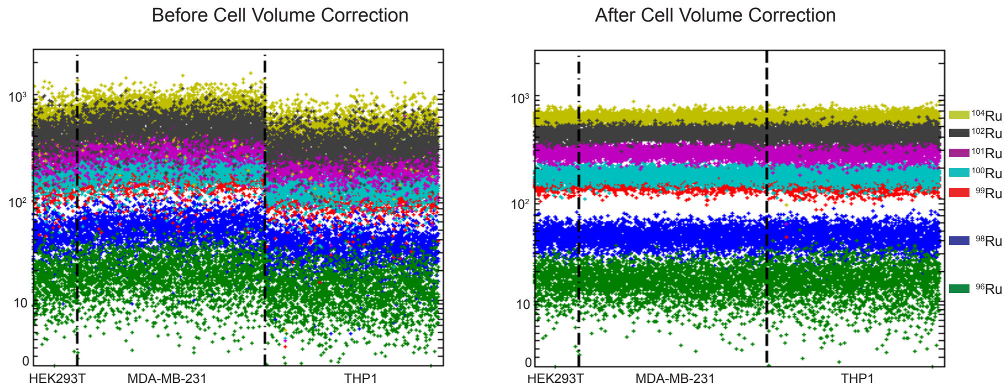

Supplementary Figure 17: **Cell volume correction applied to ASCQ_Ru measurements removes cell type variability.** Data from all seven stable isotopes of ASCQ_Ru, indicated with different colors, as measured in in HEK293T, MDA-MB-231 and THP-1 cells (left). After cell volume correction (right), the measurements are aligned vertically and no cell-line-specific variation is observed.

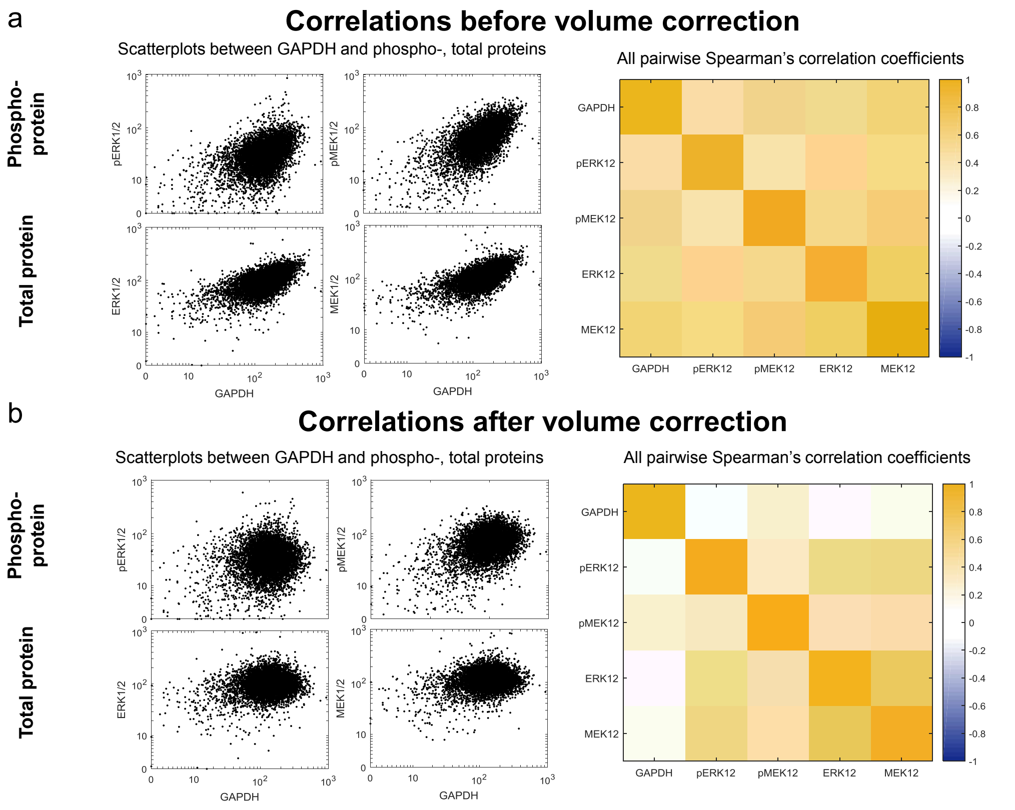

Supplementary Figure 18: **Pairwise correlation coefficients between GAPDH and MEK1/2, ERK1/2 (total and activated state), before and after cell volume correction.** We assessed the abundance of both the total amount and the phosphorylated amount for proteins ERK1/2 and MEK1/2 and we subsequently examined whether and to which extent they are affected by volume. We used the ruthenium isotopes to normalize the data and correct for volume, and quantified the housekeeping protein GAPDH as an independent validation marker (GAPDH measurements were not corrected). **(a)** Before volume correction both ERK1/2 and MEK1/2 were correlated with GAPDH (Spearmans correlation coefficients shown on the left), in both their total and the activated state. **(b)** However, after cell volume correction the effect disappeared and correlation coefficients with GAPDH were significantly lower for all proteins in both states (total and active). Within-protein correlations were preserved, however.

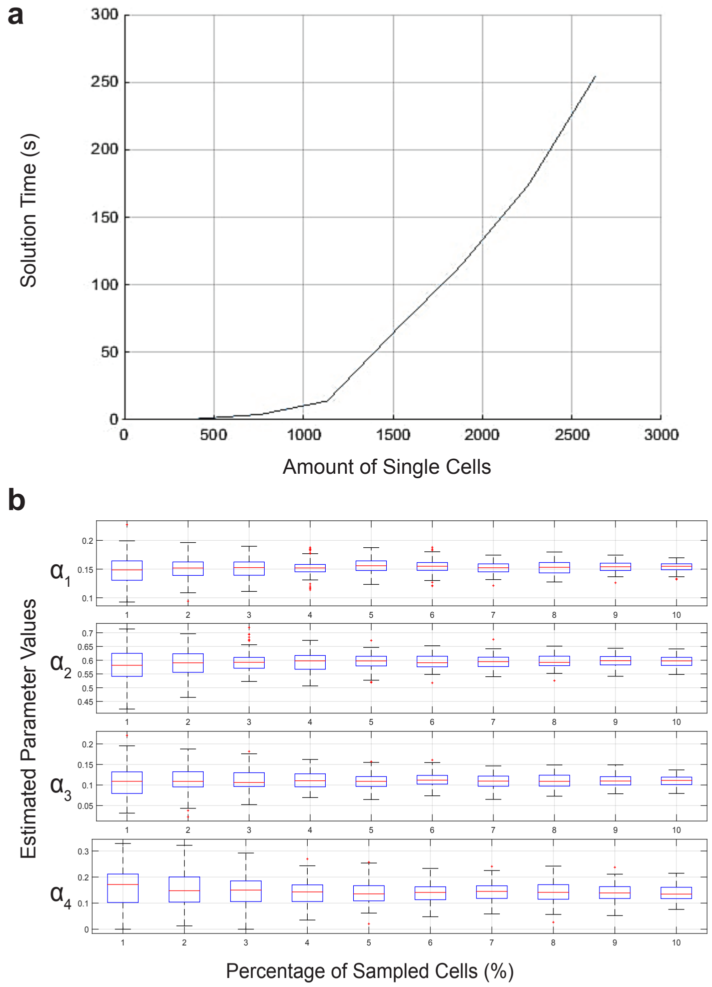

Supplementary Figure 19: **Solution time and robustness of the linear programming (LP) optimization process.** **(a)** Runtime of the LP solution with respect to the number of single cells considered. **(b)** Boxplots of distributions of estimated values of parameters α, acquired after 100 random repetitions of the optimization process, for a varying percentage of sampled single cells, indicates robust results when a minimum of 5% of the total population is sampled.

Supplementary Table 1: Antibody panel

Isotope	Antigen	Immunogen	Supplier	Clone	Staining Concentration [µg/ml]	Gene ID	UniProt Entry
La139	p-CREB/ATF1	p-Ser133 of CREB/pSer63 of ATF1	BD	J151-21	1	*CREB1*<br>*ATF1*	P16220<br>P18846
Pr141	p-STAT1	p-Tyr701	BD	4a	2	*STAT1*	P42224
Nd142	p-SRC	p-Tyr418	eBioscience	SC1T2M3	1	*SRC*	P12931
Nd143	p-FAK	p-Tyr397	CST	Polyclonal	2	*PTK2*	Q05397
Nd144	p-MEK1/2	p-Ser221	CST	166F8	0.5	*MAP2K1*<br>*MAP2K2*	Q02750<br>P36507
Nd145	p-MAPKAPK2	p-Thr334	CST	27B7	1	*MAPKAPK2*	P49137
Nd146	p-STAT5	p-Tyr694	BD	47/Stat5	2	*STAT5A*	P42229
Sm147	p-MKK4	Ser257/Thr261	CST	C36C11	1	*MAP2K4*	P45985
Nd148	p-p70S6K	p-Thr389	CST	1A5	2	*RPS6KB1*	P23443
Sm149	p-p53	p-Ser15	CST	16G8	1	*TP53*	P04637
Nd150	p-NFκB	p-Ser529	BD	K10-895.12.50	1.5	*RELA*	Q04206
Eu151	p-p38	p-Thr180/p-Tyr182	BD	36/p38	2	*MAPK14*<br>*MAPK11*<br>*MAPK12*<br>*MAPK13*	Q16539<br>Q15759<br>P53778<br>O15264
Sm152	p-AMPKα	p-Thr172	CST	40H9	1.5	*PRKAA*	Q13131
Eu153	p-AKT	p-Ser473	CST	D9E	1	*AKT1*<br>*AKT2*<br>*AKT3*	P31749<br>P31751<br>Q9Y243
Sm154	p-ERK1/2	p-Thr202/p-Tyr-204	BD	20A	1	*MAPK3*<br>*MAPK1*	P27361<br>P28482
*Sm154	cyclin E1	Recombinant human cyclin E1	CST	HE12	2	*CCNE1*	P24864

Gd156	cyclin B1	Recombinant human cyclin B1	BD	GNS-11	0.5	*CCNB1*	P14635
Gd158	p-GSK3β	p-Ser9	CST	D85E12	0.25	*GSK3B*	P49841
Tb159	GAPDH	Purified rabbit muscle GAPDH	Thermo	6C5	0.25	*GAPDH*	P04406
Gd160	p-MKK3/6	p-Ser189 of MKK3/p-Ser207 of MKK6	CST	D8E9	0.5	*MAP2K3*<br>*MAP2K6*	P46734<br>P52564
Dy161	p-PDK1	p-Ser241	BD	J66-653.44.22	0.05	*PDPK1*	O15530
Dy162	p-BTK/ITK	p-Tyr551 of BTK/p-Tyr551 of ITK	BD	24a/BTK	1	*BTK*<br>*ITK*	Q06187<br>Q08881
Dy163	p-p90RSK	p-Ser380	CST	D5D8	2	*RPS6KA1*<br>*RPS6KA2*<br>*RPS6KA3*	Q15418<br>Q15349<br>P51812
Dy164	RAB7	A synthetic peptide corresponding to residues surrounding Glu188 of human Rab7 protein	CST	D95F2	0.05	*RAB7A*	P51149
Ho165	β-catenin	Non-phospho Ser33/37/Thr41	CST	D13A1	0.5	*CTNNB1*	P35222
*Ho165	p-CDK1	Tyr15	BD	pY15	2	*CDK1*	P06493
Er166	p-STAT3	p-Tyr705	BD	4/P-STAT3	2	*STAT3*	P40763
Er167	p-JNK	p-Thr183/Tyr185	CST	G9	4	*MAPK8*<br>*MAPK9*<br>*MAPK10*	P45983<br>P45984<br>P53779
Er168	p-MARCKS	p-Ser167/170	CST	D13E4	4	*MARCKS*	P29966
Tm169	p-PLCγ2	p-Tyr759	BD	K86-689.37	1	*PLCG2*	P16885
Er170	p-HH3	p-Ser28	BD	HTA28	0.1	*H3F3A*	P68431

Yb171	p-S6	p-Ser235/Ser236	BD	N7-548	0.1	*RPS6*	P62753
Yb172	cleaved PARP	A peptide corresponding to the N-terminus of the cleavage site (Asp 214) of human PARP	BD	F21-852	2	*PARP1*	P09874
Yb173	PCNA	Recombinant rat PCNA	BioLegend	PC10	0.05	*PCNA*	P12004
Yb174	actin	A synthetic peptide corresponding to residues near the amino terminus of human β-actin protein	CST	D6A8	0.025	*ACTB*	P60709
Lu175	p-RB	p-807/811	CST	D20B12	1	*RB1*	P06400
Yb176	p-4EBP1	p-Thr37/46	CST	236B4	0.1	*EIF4EBP1*	Q13541

*Alternative antibodies for the channel Sm154 and Ho165, respectively

Supplementary Note 1 Exploring confounding factors in CyTOF data

In this section we present some illustrative examples of cell-volume and cell-cycle-induced variability and discuss how this effect can confound statistical analysis of CyTOF data. Assuming $j = 1, \ldots, m$ denotes protein markers and $i = 1, \ldots, n$ individual cells, let $y_{i,j}$ denote the raw abundance of marker j in cell i. In our CyTOF experiments, a total of $m = 31$ proteins were quantified in a population of $n \approx 3750$ THP-1 (embryonic kidney) cells. According to common practice in cytometry, the single cell raw measurements were transformed using the inverse hyperbolic sine function (asinh):

$$y_{i,j}^{trans} = \operatorname{asinh}(y_{i,j}) = \ln\left(\frac{y_{i,j}}{c} + \sqrt{\left(\frac{y_{i,j}}{c}\right)^2 + 1}\right) \qquad (1)$$

where c is a scaling factor set to 5.

Transformed single-cell measurements were manually gated according to the four cell cycle phases – G0/G1, S, G2 and M – following standard protocols [1]. We estimated Spearman correlation coefficients between pairs of proteins in each stage of the cell cycle and in ungated cells, which represent a mixed, non-synchronized population. Striking differences across phases were observed, as for instance between proteins pAMPK and pPDK1 (see scatter plot of main Fig.1a). More specifically, M phase cells are highly correlated, inducing an overall increase of the whole cell population correlation. To investigate the extend of this effect, we computed all pairwise Spearman correlation coefficients, using all cells, $\rho_{\text{all cells}}$, and G0/G1 cells, $\rho_{\text{G0/G1}}$. To compare these two groups, the correlation coefficients ρ were transformed into normally distributed variables $z(\rho)$ using the Fisher z-transformation [5]:

$$z(\rho) = \text{arctanh}(\rho) = \frac{1}{2}\ln\left(\frac{1+\rho}{1-\rho}\right) \tag{2}$$

Under this transformation, variables $z(\rho)$ follow a normal distribution with standard deviation $\sigma = \sqrt{\frac{1.06}{N-3}}$, where $N$is the number of observations. We can therefore compare any two correlations ρ_1, ρ_2 estimated from two sets containing N_1, N_2 observations using a t-test:

$$Z(\rho_1, \rho_2) = \frac{z(\rho_1) - z(\rho_2)}{\sqrt{\frac{1.06}{N_1-3} + \frac{1.06}{N_2-3}}} \tag{3}$$

We estimated the p-values of all Z-scores at a significance level a of 0.05. The results are shown graphically in the heatmap of Supplementary Fig. 1a. Most pairs of proteins show a significant change in correlation computed using all cells versus correlations computed using G0/G1 cells. To determine whether this effect is due to the bias introduced by M phase cells, we excluded the data of these cells and repeated the analysis. The results are shown in the heatmap of Supplementary Fig.1b; the effect is less pronounced when data on M phase cells is excluded, but for a high percentage of protein pairs, correlations are significantly different.

To investigate whether the cell volume has a similar effect, cells in G0/G1 phase were manually gated and separated in three categories based on their size, indicated by ASCQ_Ru staining. Similarly as above, Spearman correlation coefficients between pairs of proteins in each category ($\rho_{\text{small}}, \rho_{\text{intermediate}}, \rho_{\text{big}}$) and in the whole population of cells ($\rho_{\text{all cells}}$) were compared. The results are shown graphically in the heatmaps of Supplementary Fig. 4. We observe that most pairs of proteins show a statistically significant change in correlation coefficients computed using all cells versus correlation coefficients computed using small or in-

termediate cells.

Supplementary Note 2 Cell volume correction

We describe here how the ASCQ_Ru measurements are used for cell volume correction. Reusing previous notation, let $y_{i,j}$ denote the raw abundance of marker j in cell i, where $i = 1, \ldots, n$ and $j = 1, \ldots, m$. Let $v = 1, \ldots, l$ denote the cell volume marker index, with $l < m$. Initially, the raw cell volume measurements $y_{i,v}$ are normalized by dividing by the mean value for each cell volume marker v across all cell lines:

$$y_{i,v}^{norm} = \frac{y_{i,v}}{\frac{1}{n}\sum_{i=1}^{n} y_{i,v}}$$

The resulting measurements are averaged across all markers, resulting in a normalization factor vol_i:

$$\text{vol}_i = \frac{1}{l}\sum_{v=1}^{l} y_{i,v}^{norm}$$

We use the mean of all cell volume markers, as vol_i is less noisy, less likely to contain zero elements and a more robust indication of cell volume that the individual markers. Lastly, raw measurements of all markers j are divided by vol_i to correct for volume-induced variability:

$$y_{i,j}^{corr} = \frac{y_{i,j}}{\text{vol}_i}$$

The results of the volume correction step are shown graphically in Supplementary Fig.17. We observe that Ruthenium measurements from three different cell lines almost perfectly align after the correction step.

Supplementary Note 3 Cell cycle classification

Decision tree implementation

In this section we provide a detailed description of the implementation of the decision tree and its performance on experimental CyTOF data. Let $p \in \{1, \ldots, 4\}$ represent the class label, in our case the cell cycle phase index (1: G0/G1, 2: S, 3: G2 and 4: M). For the purposes of training the decision tree, we used existing measurements of four well-established cell cycle markers IdU, cyclin B1, p-HH3 and p-RB, from a THP-1 cell line together with cell class labels, derived by manual gating in Cytobank (see Supplementary Fig. 6 (a)). Prior to the training, the data was transformed and standardized using eqs. 1 and 5 and then randomly split into training and testing sets, in the proportions of 70% and 30%, respectively. During the learning phase, the algorithm examines the training data together with the class labels and identifies an optimal split that minimizes a predefined optimization criterion. In our implementation, we chose the Gini diversity index (*gdi*) as optimization criterion:

$$gdi = 1 - \sum_{p=1}^{4} r_p^2, \tag{4}$$

where r_p denotes the observed proportion of single-cell measurements belonging to phase p. A pure node (i.e., a node that contains observations belonging to only one class) has a *gdi* equal to zero, and all non-pure nodes have $gdi > 0$. The partitioning continues in the children nodes until one of two termination conditions is fulfilled: 1) a node is pure or 2) the putative new split produces leaves with fewer observations than a specified threshold, set here to 10. After the training is finalized, a class index is assigned to each terminal node, according to the prevalent class in the leaf (i.e., the class with the highest proportion

of observations). The resulting decision tree is shown in Supplementary Fig.6b below. In our case, all terminal nodes were pure, indicating a 100% accuracy on the training set. Similarly, accuracy on the test set was also 100%. We then examined whether the trained classifier produced accurate predictions using measurements from other cell lines. Results for HEK293T cells are shown in Supplementary Fig.7a in the form of a confusion matrix, where rows correspond to the actual class of the samples, assigned through manual gating, and columns to the class predicted by the decision tree. Elements in the diagonal correspond to the number of correctly classified samples per class and elements away from the diagonal correspond to elements wrongly classified. Using the decision tree G0/G1, S and M classification performance exceeded 97.5%; for G2 phase performance was marginally lower at 88.3%. While the classification performance is satisfactory, we show in the next section how it can be improved by using Gaussian mixture models in conjunction with decision trees.

Fitting Gaussian mixture models to the measurements

Gaussian mixture models (GMMs) are probabilistic models that aim to represent a sample population as a mixture of Gaussian subpopulations, each characterized by different mean vectors and covariance matrices [7]. The definition is formalized as follows: let $Y = \{y_1, \ldots, y_n\}$ denote the four-dimensional data matrix of the cell cycle markers, transformed and standardized according to eqs. 1. To eliminate any systematic bias introduced by differences in antibody concentrations or affinities prior to the analysis, the measurements were standardized as follows:

$$y_{i,j}^{stand} = \frac{y_{i,j}^{trans} - \bar{y}_j^{trans}}{s_j^{trans}} \tag{5}$$

where $\bar{y}_j^{trans}$ and s_j^{trans} denote the sample mean and standard deviation of each marker respectively, computed across all cells and time points.

A GMM with parameters θ is defined as follows:

$$p(Y|\theta) = \sum_{p=1}^{4} w_p \, \mathcal{N}(y|\mu_p, \Sigma_p) \tag{6}$$

where each $\mathcal{N}(Y|\mu_p, \Sigma_p)$ is a component of the model, defined as a four-dimensional Gaussian density with mean vector μ_p and covariance matrix Σ_p:

$$\mathcal{N}(Y|\mu_p, \Sigma_p) = \frac{1}{\sqrt{(2\pi)^4|\Sigma|}} \exp\left(-\frac{1}{2}(Y-\mu_p)' \, \Sigma_p^{-1} \, (Y-\mu_p)\right), \tag{7}$$

and w_p are the mixture coefficients (proportions of each component), that satisfy $w_p \geq 0$ and $\sum_{p=1}^{4} w_p = 1$. Each mixture p is characterized by the parameters θ:

$$\theta = \{w_p, \mu_p, \Sigma_p\}, \quad p = 1, \ldots, 4. \tag{8}$$

The optimal parameters θ to fit the single-cell measurements Y are computed through maximum likelihood (ML) estimation using an iterative expectation-maximization (EM) process, where θ is initialized using the output of the decision tree classifier. This biases the GMM towards converging to the solution of the decision tree, while overcoming the problem of the rigidity of the decision tree's boundaries. The EM process is repeated until convergence, achieved when the log-likelihood function fails to decrease more than 10^{-6}, or until a maximum number of 100 iterations is reached (termination without convergence). Results of this analysis on data from HEK293T cells are shown in Supplementary Fig.7b in the form of a confusion matrix. This analysis demonstrates that GMMs improve the classification performance achieved by decision trees. The gain in performance

arises from the GMM probabilistic treatment of class assignments. An outlier cell has a non-zero probability to be assigned to its real class, even though according to the DT should have been assigned to a different class.

Supplementary Note 4 Cell cycle trajectory

In this section we provide details about the trajectory reconstruction method. Let y_i denote the four-dimensional vector of cell cycle markers (IdU, cyclin B1, p-HH3 and p-RB) and let $p_i \in \{1, ..., 4\}$ denote the cell cycle phase of each single cell i. Next, let $f_\alpha(y)$ denote a linear function that embeds the four-dimensional space of the cell cycle markers and projects them into a one-dimensional space, which in our case represents biological pseudotime:

$$f_\alpha(y_i) = \sum_{j=1}^{4} \alpha_j \, y_{i,j} \, , \tag{9}$$

where the vector of coefficients of the linear terms $\alpha = (\alpha_1 \; \alpha_2 \; \alpha_3 \; \alpha_4)$ takes values in $\mathbb{R}^4_{\geq 0}$. In the following, we define a linear optimization problem that identifies the optimal coefficients α to define the biological pseudotime. Let $C_p = \{i \,|\, p_i = p\}$ denote the subpopulation of cells in phase $p = 1, ..., 4$. Our problem is then to find coefficients α such that the ordering of the classes is preserved, that is:

$$p < q \;\Rightarrow\; f_\alpha(y_{i_p}) < f_\alpha(y_{i_q}) \;\; \forall \;\; i_p \in C_p, i_q \in C_q \, . \tag{10}$$

By exploiting transitivity and since the order of the classes is known, we can reduce the number of constraints and impose only order preservation between consecutive classes, i.e. for $q = p+1$. Thus the subsets of cell pairs on which we impose constraints are defined as:

$$S_p = \{(i_1, i_2) \,|\, i_1 \in C_p, \, i_2 \in C_{p+1}\} \, , \, p = 1, ..., 3.$$

Enforcing the ordering constraints for all cells in adjacent classes might be in general infeasible due the high level of noise of CyTOF data. Therefore, the inequality constraints among cells

i_1, i_2 belonging to S_p, $p = 1, ..., 3$, are redefined by introducing slack variables s_{i_1,i_2}, positive variables that allow the violation of the constraints. In this context, a slack variable represents the degree of constraint violation, and our optimization problem is translated to estimating α so that the sum over all slack variables is minimized (Supplementary Fig. 8), formulated as the following linear program (LP):

$$\min_{\substack{\alpha \in \mathbb{R}^4_{\geq 0} \\ s_{i_1,i_2} \geq 0,\ (i_1,i_2) \in S_p \\ p=1,...,3}} \sum_{p=1}^{3} \sum_{(i_1,i_2)\in S_p} s_{i_1,i_2} \tag{11}$$

$$\text{subject to: } f_\alpha(y_{i_1}) \leq f_\alpha(y_{i_2}) + s_{i_1,i_2} \quad \forall (i_1, i_2) \in S_p,\ p = 1, ..., 3.$$

The LP stated in eq.11 has a trivial solution (i.e., $\alpha = 0$). In order to exclude this trivial solution, we impose the following constraint:

$$\sum_{j=1}^{4} \alpha_j = 1. \tag{12}$$

The choice of the right-hand-side of eq.12 is arbitrary. A different positive choice would only amount to a scaling of the LP, leaving the qualitative results unchanged.

Slack penalization on cell pairs from adjacent classes implicitly puts an over-proportional weight on larger classes, in particular on adjacent larger classes, due to the imbalance in the number of constraints. Thus, we add weights to the objective function to obtain a balanced result. The weight for each pair of adjacent classes is given as:

$$w_p = \frac{1}{\sqrt{|C_p| \cdot |C_{p+1}|}}. \tag{13}$$

Together with eq.12 this leads to the following modified version of the LP presented in eq.11:

$$\begin{aligned}
\min_{\substack{\alpha \in \mathbb{R}^4_{\geq 0} \\ s_{i_1,i_2} \geq 0,\ (i_1,i_2) \in S_p \\ p=1,...,3}} \quad & \sum_{p=1}^{3} w_p \sum_{(i_1,i_2)\in S_p} s_{i_1,i_2} \\
\text{subject to: } & f_\alpha(y_{i_1}) \leq f_\alpha(y_{i_2}) + s_{i_1,i_2} \quad \forall (i_1,i_2) \in S_p,\ p = 1,...,3 \\
& \sum_{j=1}^{4} \alpha_j = 1.
\end{aligned} \tag{14}$$

Depending on the number of cells considered, as shown in Supplementary Fig.19a the running time to solve the resulting LP can be very large and thus the problem becomes computationally intensive. To reduce the solution time, we randomly pick a fixed percentage of cells from each class (C_p, $p = 1, ..., 4$) and adjust the weights w_p accordingly. To investigate the robustness of the solution with respect to the sampling, we solved the LP 100 times with randomly picked cells and repeated this for an increasing total number of cells. Results are summarized in Supplementary Fig.19b. The resulting solutions - the values of parameters α - are robust to the sampling of cells even for a small percentage of cells (>5%). Once parameters α are identified, all single cells $i = 1, \ldots, n$ are arranged on the new pseudotemporal dimension by sorting them in an ascending order based on the value of $f_a(y_i)$, resulting in a new ordering index i_s. Then, the corresponding cell cycle trajectories for each marker $j = 1, \ldots, m$ are denoted as $y_{i_s,j}$.

Supplementary Note 5 Cell cycle alignment

In this section we provide further details on how the relative cell cycle phase proportions across individual samples can be aligned ain order to correct for cell cycle phase duration variability. This correction does however imply downsampling the data, which modifies the initial data distribution, and thus should be used cautiously and only for the purpose of comparing data across different cell lines.

Let $p \in \{1, \ldots, 4\}$ represent the cell cycle phase (1: G0/G1, 2: S, 3: G2 and 4: M). Then, let $k = 1, \ldots, K$ denote the different samples, n_p^k the number of cells in sample k and phase p and r_p^k denote the observed proportion of single-cell measurements belonging to phase p in sample k, i.e. $r_p^k = \frac{n_p^k}{\sum_{p=1}^4 n_p^k}$. To align the cell cycle phase proportions across all samples, we need to equalize all individual r_p^k to some common target proportions $\bar{r}_p$, in our case selected either as the mean proportions across samples or as the proportions of one reference sample. This is achieved by a downsampling strategy, which aims to equalize the class proportions while at the same time preserving the maximum amount of single cell data. More specifically, for each sample k the following steps are executed:

1. Estimate a sampling indicator λ_p^k that represents the proportion of cells to be removed from sample k in phase p in order to meet the target proportions:

$$\lambda_p^k = \frac{n_p^k - \bar{r}_p \sum_{p=1}^4 n_p^k}{n_p^k} \tag{15}$$

   A negative value of λ_p^k in eq.15 indicates that the existing number of cells in that phase is not sufficient to match the target proportions $\bar{r}_p$ by downsampling.

2. Identify the index of phase p_m with the lowest indicator:

$$\lambda_{p_m}^k = \min \lambda_p^k$$

3. The number of samples $\bar{n}_p^k$ to be drawn from each phase p in order to satisfy the target proportions is computed as follows:

$$\bar{n}_p^k = n_{p_m}^k \left(\frac{\bar{r}_p}{r_{p_m}^k} \right)$$

An example of the alignment results is shown graphically in Supplementary Fig.13 for protein p-RB in HEK 293T cells.

Supplementary Note 6 Cell cycle correction

After (optionally) equalizing cell cycle phase duration, the last step is to remove cell cycle fluctuations, correcting for unwanted variability. Reusing the notation of Supplementary Note 4, let the single cell trajectory of each marker j be $y_{i_s,j}$. To correct for cell cycle variations, the following process is executed for all markers $j = 1, \ldots, m$:

1. Compute the mean trajectory $\bar{y}_{i_s,j}$ by applying a mean filter on $y_{i_s,j}$, where the value of each single cell i_s is replaced by the mean of the neighboring cells in a window of fixed size.

2. Normalize $\bar{y}_{i_s,j}$ by dividing it with its mean value, i.e.:

$$\bar{y}_{i_s,j}^{norm} = \frac{\bar{y}_{i_s,j}}{\frac{1}{n}\sum_{i=1}^{n} \bar{y}_{i_s,j}}$$

3. Divide the single cell trajectory with the normalized mean trajectory:

$$y_{i_s,j}^{corr} = \frac{y_{i_s,j}}{\bar{y}_{i_s,j}^{norm}}$$

In this way, cell-cycle-specific fluctuations are removed and the single cells are redistributed independently of cell cycle variation.

References

[1] Gregory K. Behbehani et al. "Single-cell mass cytometry adapted to measurements of the cell cycle". In: *Cytometry Part A* 81.7 (2012), pp. 552–566. URL: http://onlinelibrary.wiley.com/doi/10.1002/cyto.a.22075/full (visited on 04/14/2016).

[2] Sean C. Bendall et al. "Single-cell trajectory detection uncovers progression and regulatory coordination in human B cell development". eng. In: *Cell* 157.3 (Apr. 2014), pp. 714–725. ISSN: 1097-4172. DOI: 10.1016/j.cell.2014.04.005.

[3] Laleh Haghverdi, Florian Buettner, and Fabian J. Theis. "Diffusion maps for high-dimensional single-cell analysis of differentiation data". In: *Bioinformatics* 31.18 (Sept. 2015), pp. 2989–2998. ISSN: 1367-4803. DOI: 10.1093/bioinformatics/btv325. URL: https://academic.oup.com/bioinformatics/article/31/18/2989/241305/Diffusion-maps-for-high-dimensional-single-cell (visited on 08/30/2017).

[4] Zhicheng Ji and Hongkai Ji. "TSCAN: Pseudo-time reconstruction and evaluation in single-cell RNA-seq analysis". In: *Nucleic Acids Research* 44.13 (July 2016), e117–e117. ISSN: 0305-1048. DOI: 10.1093/nar/gkw430. URL: https://academic.oup.com/nar/article/44/13/e117/2457590/TSCAN-Pseudo-time-reconstruction-and-evaluation-in (visited on 08/30/2017).

[5] M. G. Kendall, A. Stuart, and J. K. Ord, eds. *Kendall's Advanced Theory of Statistics*. New York, NY, USA: Oxford University Press, Inc., 1987. ISBN: 978-0-19-520561-9.

[6] Eugenio Marco et al. "Bifurcation analysis of single-cell gene expression data reveals epigenetic landscape". en. In: *Proceedings of the National Academy of Sciences* 111.52 (Dec. 2014), E5643–E5650. ISSN: 0027-8424, 1091-6490. DOI: 10.1073/pnas.1408993111. URL: http://www.pnas.org/content/111/52/E5643 (visited on 08/30/2017).

[7] Geoffrey McLachlan and David Peel. *Finite mixture models*. John Wiley & Sons, 2004. URL: https://books.google.ch/books?hl=en&lr=&id=c2_fAox0DQoC&oi=fnd&pg=PR7&dq=Finite+Mixture+Models&ots=IsTB1-83qv&sig=A6IHz9dLLkhZBxeVN6Xww0nwuN0 (visited on 05/09/2016).

[8] Cole Trapnell et al. "The dynamics and regulators of cell fate decisions are revealed by pseudotemporal ordering of single cells". en. In: *Nature Biotechnology* 32.4 (Apr. 2014), pp. 381–386. ISSN: 1087-0156. DOI: `10.1038/nbt.2859`. URL: `https://www.nature.com/nbt/journal/v32/n4/full/nbt.2859.html` (visited on 08/30/2017).
